# Wnt/β-Catenin Signaling Enhances Cyclooxygenase-2 (COX2) Transcriptional Activity in Gastric Cancer Cells

**DOI:** 10.1371/journal.pone.0018562

**Published:** 2011-04-06

**Authors:** Felipe Nuñez, Soraya Bravo, Fernando Cruzat, Martín Montecino, Giancarlo V. De Ferrari

**Affiliations:** 1 Centro de Tecnología e Innovación para el Cáncer (CTI-Cáncer), Department of Biochemistry and Molecular Biology, Faculty of Biological Sciences, Universidad de Concepción, Concepción, Chile; 2 Center for Biomedical Research, Faculty of Biological Sciences and Faculty of Medicine, Universidad Andrés Bello, Santiago, Chile; Roswell Park Cancer Institute, United States of America

## Abstract

**Background:**

Increased expression of the cyclooxygenase-2 enzyme (COX2) is one of the main characteristics of gastric cancer (GC), which is a leading cause of death in the world, particularly in Asia and South America. Although the Wnt/β-catenin signaling pathway has been involved in the transcriptional activation of the COX2 gene, the precise mechanism modulating this response is still unknown.

**Methodology/Principal Findings:**

Here we studied the transcriptional regulation of the COX2 gene in GC cell lines and assessed whether this phenomenon is modulated by Wnt/β-catenin signaling. We first examined the expression of COX2 mRNA in GC cells and found that there is a differential expression pattern consistent with high levels of nuclear-localized β-catenin. Pharmacological treatment with either lithium or valproic acid and molecular induction with purified canonical Wnt3a significantly enhanced COX2 mRNA expression in a dose- and time-dependent manner. Serial deletion of a 1.6 Kbp COX2 promoter fragment and gain- or loss-of-function experiments allowed us to identify a minimal Wnt/β-catenin responsive region consisting of 0.8 Kbp of the COX2 promoter (pCOX2-0.8), which showed maximal response in gene-reporter assays. The activity of this pCOX2-0.8 promoter region was further confirmed by site-directed mutagenesis and DNA-protein binding assays.

**Conclusions/Significance:**

We conclude that the pCOX2-0.8 minimal promoter contains a novel functional T-cell factor/lymphoid enhancer factor (TCF/LEF)-response element (TBE Site II; -689/-684) that responds directly to enhanced Wnt/β-catenin signaling and which may be important for the onset/progression of GC.

## Introduction

Gastric cancer (GC) is a multifactorial disease, characterized by highly malignant neoplasms in the gastric mucosa, and represents the second leading cause of cancer death worldwide with the highest prevalence in Asia and South America [1], [2], [3]. Environmental associated risk factors include diet, snuff consumption, obesity and *Helicobacter pylori* infection [4]. Several mutations in tumor-suppressor genes, including P53, adenomatous polyposis coli (APC), E-cadherin and RUNX3 [3], [5], as well as in oncogenes like k-ras, HER2 and β-catenin [3], [6], [7], have been documented in GC. In addition, over expression of various genes has been documented, including WNT2B [8], TC1 (C8orf4) [9], and the cyclooxygenase 2 (COX2) enzyme, which catalyzes the crucial step in the production of prostaglandin E2, a key mediator of joint inflammation [10], [11].

It has been observed that the expression of the COX2 gene is significantly increased in human gastric adenocarcinoma tissues, when compared with paired gastric mucosal specimens devoid of cancer cells [10]. Such increased expression has been proposed to affect the intensity of invasion, size, lymph node metastases, tumor development and bad prognosis [4], [12], [13]. In this regard, large amounts of data describe chemo-preventive and anticancer activity of non steroidal anti-inflammatory drugs (NSAID) including selective COX2 inhibitors as potential treatments for GC [10], [11], [14].

Transcriptional control of the COX2 gene depends on the molecular machinery interacting with the COX2 promoter, which seems to be controlled through the activity of various signaling pathways [15], [16], [17]. Indeed, it was initially established that CRE (−59/−53), NF-IL6 (−132/−124) and NF-κB (−233/−214) consensus sequences in the COX2 promoter were necessary for the expression of the gene [16]. Subsequent functional studies in the COX2 promoter identified a series of regulatory elements participating in the transcription of the gene, including AP-1, AP-2, Sp-1, C/EBPβ [18], [19], [20] and proteins belonging to the T-Cell factor/Lymphoid enhancer factor (TCF/LEF) family of transcription factors, which are crucial for Wnt/β-catenin signal transduction [21].

The Wnt/β-catenin signaling pathway is widely acknowledged as playing a major role in human disease, particularly in the onset and development of cancer [22], [23], [24]. Interestingly, recent experiments in GC derived cells have shown a relationship between COX2 expression and the inhibition of the Glycogen Synthase Kinase-3β (GSK3β) enzyme [25], which is a key Wnt component that phosphorylates β-catenin and promotes its subsequent degradation via proteasome [26]. The relationship between Wnt/β-catenin and COX2 expression in different cancer cell models is further supported from the following studies. First, it has been observed in the mammary epithelium that Wnt/β-catenin play an indirect effect on COX2 transcription, which could be mediated by up-regulation of an intermediary factor PEA3 [27]. Second, and in contrast to an indirect mode of action, Araki and cols. [21] reported that in colon cancer cells there is an induction in COX2 expression through a β-catenin/TCF dependent mechanism, and partially characterized a consensus TCF/LEF binding site (TBE: core CTTTG) positioned 1,079 bp upstream from the transcriptional start site in the COX2 promoter. Third, it was observed that in colon cancer patients and derived cell lines there is an association between overexpression of the Wnt pathway-associated proteins LEF-1 and Pontin52/TIP49a and up-regulation of COX2 expression [28]. Finally, using chondrocytes, it has been demonstrated that LEF-1, together with β-catenin, regulated COX2 expression by direct binding of the LEF-1/β-catenin complex to the 3′UTR region of the COX2 genomic locus [29]. Therefore, at present there is not a clear picture as to whether Wnt signaling is involved in COX2 gene expression, or as its role in GC onset/progression. Here we sought to understand whether there is a direct regulation of the COX2 gene expression via Wnt/β-catenin signaling and to identify Wnt/β-catenin regulatory elements in the promoter region of the COX2 gene which can upregulate COX2 transcription in GC cells.

## Materials and Methods

### Cells and culture conditions

Human cell-lines MKN45 (Japanese Collection of Research Bioresources, Japan), N87, SNU1, SNU16, KATOIII, AGS, WI38 and HEK293 (American Type Culture Collection; Rockville, MD) were used in this study. MKN45, N87, SNU1 and KATOIII cells were grown in RMPI media (Gibco); AGS in F12K medium (Hyclone); WI38 in EMEM (Gibco); and HEK293 in DMEM (Gibco). Culture media was supplemented with 10% FBS (Gibco) (AGS with 20%) and 1% penicillin/streptomycin. Cell-lines were maintained at 37°C in 5% CO_2_ and saturated humidity.

### Plasmids and site-directed mutagenesis

The SuperTOPFlash-luciferase and the pRL-TK renilla-luciferase plasmids [30], the constitutive active β-catenin (S33Y) [31] and the dominant-negative ΔTCF4 expression plasmids [32] have been described previously. Chimeric COX2 promoter-luciferase fragments were generated by PCR from human genomic DNA using specific primers containing restriction sites and subsequently inserted into the pGL3-Basic vector (Promega). The mutations in the TBE-II site (−689/−684) of the COX2 promoter were generated using primers pCOX-0,8-TBEMUT with the QuickChange site-directed mutagesis kit (Stratagene). Constructs were verified through direct sequencing (ABI-3130 Genetic Analyzer, Applied Biosystems). Primers sequences are described in Table S1.

### Semiquantitative and Real Time RT-PCR

Total RNA was extracted in RNAse free conditions using TRIZOL (Invitrogen) and 2 µg of RNA was reverse transcribed with 200 U of SuperScript II Reverse Transcriptase (RT) (Invitrogen) using 500 ng of Oligo(dT) primers. Experimental determination of COX2, c-myc and CCND1 mRNA levels was performed according to [33]. Briefly, cDNAs were subjected to Real-Time PCR in an iCycler iQ System (Bio-Rad Laboratories). Each 25 µl reaction volume contained 1 unit of Platinum Taq DNA polymerase (Invitrogen), 1X reaction buffer (20 mM Tris-HCl pH 8.4, and 50 mM KCl), 1.5 mM MgCl_2_, 2.5 µg BSA, 0.01% Glycerol, 200 µM of dNTPs, 0.3X SYBR Green solution and 0.4 µM of specific primers (see Table S1). PCR conditions were set as follows: 90 seconds at 94°C and then 30 cycles of 30 seconds at 94°C, 30 seconds at 62°C and 30 seconds at 72°C. All reactions were performed in triplicate and results obtained for each gene were normalized to those obtained in parallel with β-actin. The quality of RNA and PCR products was monitored throughout the experiments via electrophoresis on 1% agarose gels, stained with ethidium bromide.

### Induction of the Wnt/β-catenin signaling pathway

Wnt signaling was pharmacologically induced in MKN45 cells seeded in 6 well culture plates at 80–90% of confluence (i.e. 1, 2, 4 and 8 h of incubation) either with 10–20 mM of LiCl (Sigma) or 5–10 mM of valproic acid (VA; Sigma) as previously described [34], [35]. Then, cells were collected for mRNA extraction and real time-PCR determination as described above. Similarly, MKN45 cells were stimulated for 2 hours with 200 and 400 ng/ml of purified Wnt3a protein. Purification of Wnt3a was carried out as described [36].

### Transcriptional activity of the COX2 promoter

Activity of the COX2 promoter was measured in 80–90% confluent MKN45, AGS, WI38 and HEK293 cells seeded in 6 well culture plates. Briefly, cells were co-transfected using FUGENE (Roche) for 24 with the pCOX2-luciferase or the pSuperTOPFlash reporters and either constitutive active β-catenin (S33Y) [31] or the dominant-negative ΔTCF4 [32] constructs. The pRL-TK renilla luciferase plasmid was used as an internal control. Firefly and renilla luciferase activities were determined using the Dual-Luciferase Reporter Assay (Promega) in a Victor-3 multiplate reader instrument (Perkin Elmer), as described previously [30]. Relative luciferase activities were expressed by dividing firefly luciferase activity with renilla luciferase activity for each sample (N = 3, each in triplicate).

### Chromatin immunoprecipitation (ChIP) assays

ChIP studies were performed in MKN45 cells as described earlier [37]. The fraction of nuclear β-catenin bound either to TBE Sites I, II, III or IV in the human COX2 promoter was immunoprecipitated with anti β-catenin antibodies (Santa Cruz) and assessed by real time-PCR using specific primers (Table S1). Additionally, the fraction of the RNA polymerase II (Pol-II) and acetylated histones H3 and H4 (H3ac and H4ac) bound either to the TBE-II region (−793/−594) or the proximal region (−118/+62) of the COX2 promoter was similarly assessed (anti-Pol-II, Santa Cruz; H3ac and H4ac, Upstate). As a positive control we used the c-myc TBE site [38]. Antibody specificity was assayed with normal rabbit-IgG (Santa Cruz).

### Electrophoretic mobility shift assay (EMSA)

EMSA was performed using oligonucleotides containing either the wild-type COX2 TBE-II consensus sequence (see Table S1) or previously reported mutant TBE sequences [39]. In brief, wild-type and mutated ^32^P-labeled oligonucleotides were incubated in binding buffer with nuclear extracts of MKN-45 cells for 30 min at 30°C. Subsequently, DNA-protein complexes were separated from free oligonucleotides on a 5% non denaturating polyacrylamide gel. After electrophoresis, the gel was dried, and exposed to film for 1 day. The visualization of radioactive bands was analyzed by autoradiography. Binding specificity was checked by incubating the DNA-protein complexes in the presence of an excess of non-labeled wild-type or mutated oligonucleotides [21], [39].

### Statistical analysis

Each experiment was repeated at least three times with three replicates. Data are shown as the mean ± SD. Multiple group comparisons were performed by one-way ANOVA using the STATISTICA 9.0 software. *P*<0.05 was considered significant.

## Results

### COX2 expression correlates with nuclear β-catenin levels in GC cells

**Figure 1:**
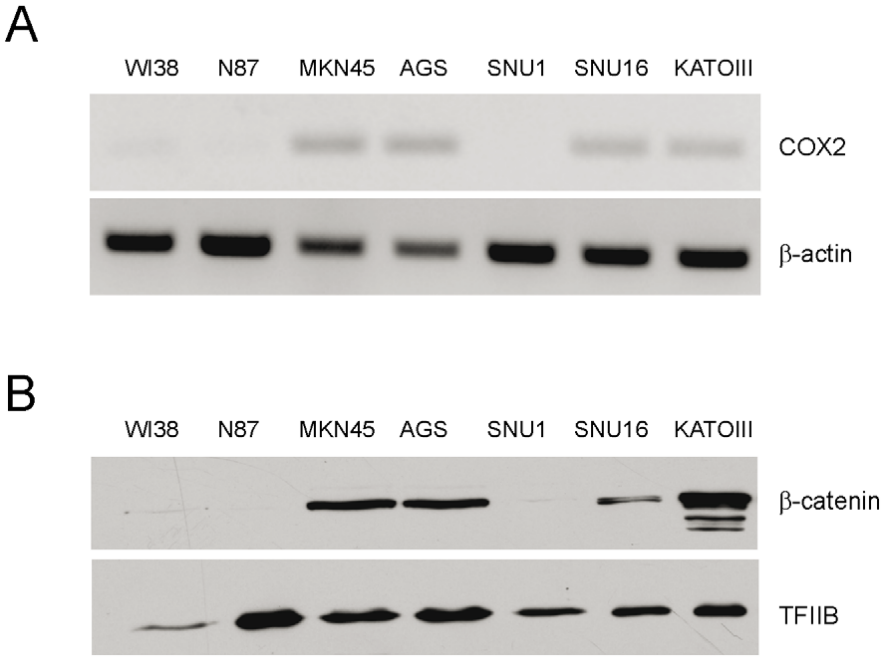
COX2 gene expression and nuclear localization of β-catenin in GC cells. (A) COX2 mRNA expression in control (WI38) and GC cell-lines (MKN45, AGS, SNU1, SNU16, KATOIII and N87). Total RNA was extracted from cultured cells and semiquantitative RT-PCR was used to determine COX2 and β-actin RNA levels as an internal control. Twenty-six cycles were chosen as an adequate PCR cycle. (B) Nuclear levels of β-catenin protein in the same cell-lines, as shown in (A), were examined through Western Blot analysis using nuclear extracts. The TFIIB general transcription factor was used as an internal control.

We initially determined the expression levels of COX2 mRNA in human GC cell-lines MKN45, N87, SNU1, SNU16, KATOIII and AGS [40], [41], [42], as well as WI38 fibroblasts used here as a control cell line [43], examining whether they were correlated with Wnt/β-catenin signaling. As depicted in Figure 1, strong levels of COX2 expression were observed as early as 26 cycles of PCR amplification in metastatic cell lines MKN45, SNU16 and KATOIII, and also in the AGS cell line that is derived from a primary tumor. This result is in agreement with COX2 expression levels detected previously in MKN45, KATOIII and AGS cells [44], [45], [46]. In contrast, COX2 mRNA levels were either very low in WI38 fibroblasts or undetectable in N87 and SNU1 cells (Figure 1A), suggesting that this differential pattern of expression is not related to metastatic stages or the level of cell transformation. Remarkably, nuclear β-catenin levels were closely related with COX2 expression, since high levels of the protein were observed in MKN45, AGS, SNU16 and KATOIII cells, compared with N87, SNU1 and WI38 cells (Figure 1B), implying a role for Wnt signaling in COX2 mRNA expression in GC cells.

### Enhancement of COX2 expression via Wnt/β-catenin signaling

**Figure 2:**
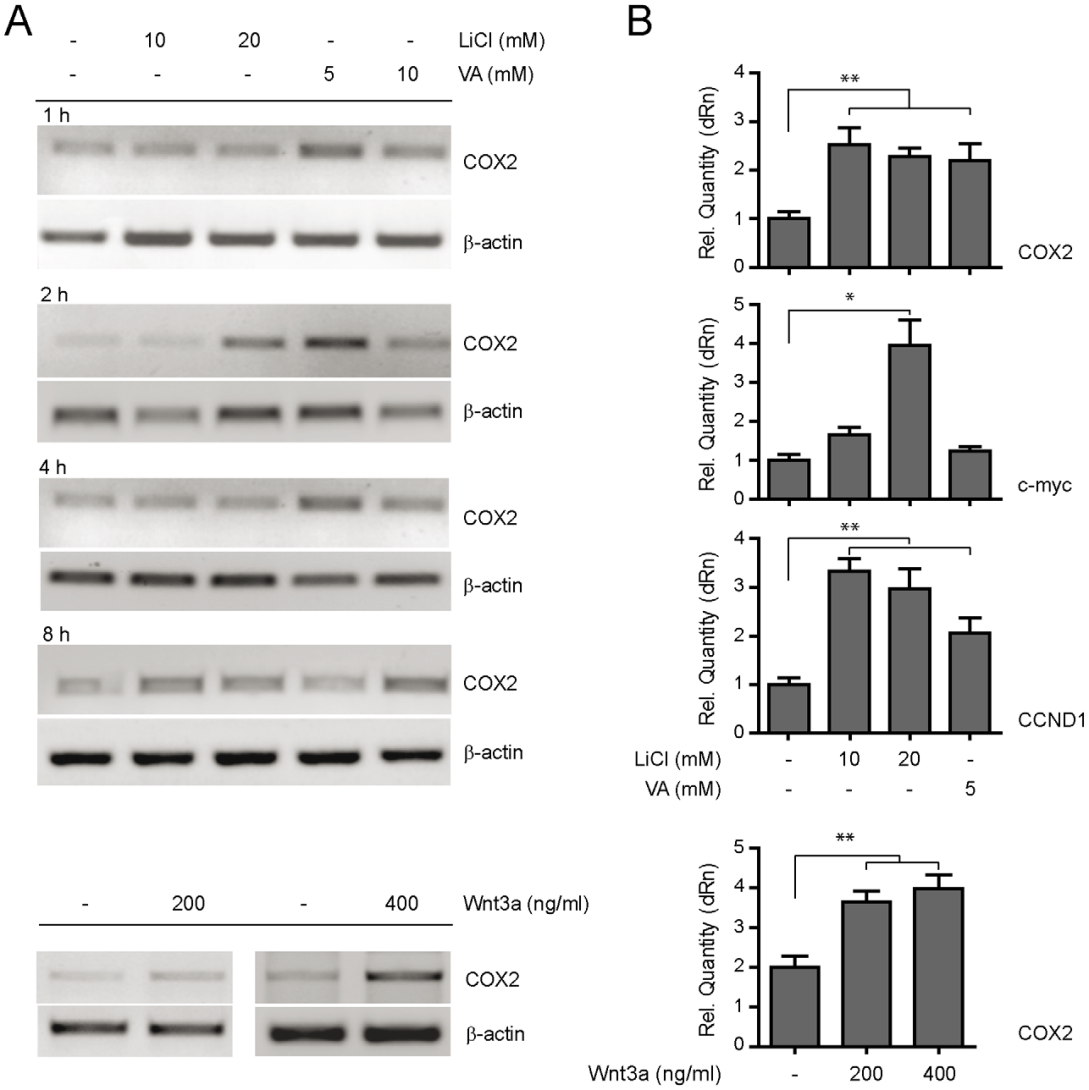
Pharmacological and molecular enhancement of COX2 expression via Wnt/β-catenin signaling. (A) COX2 mRNA expression levels in MKN45 cells treated for 1 to 8 h with either 10–20 mM of lithium (LiCl) or 5–10 mM of Valproic acid (VA) were evaluated through RT-PCR. As an internal control, β-actin levels were determined. (B) Q-PCR for COX2, c-myc and CCND1 mRNA expression stimulated pharmacologically with lithium (LiCl) and valproate (VA) during 2 h (top panel). Bottom panel, Q-PCR for COX2 in MKN45 cells stimulated with purified Wnt3a for 2 h. Q-PCR results were expressed as Relative Quantity (dRn) and β-actin mRNA levels were used as the internal control. Each figure corresponds to at least 3 independent experiments. Statistical significance was determined through ANOVA test (* p<0.05, ** p<0.01).

In order to examine if the Wnt cascade is involved in COX2 expression, MKN45 cells were pharmacologically stimulated for different periods of time (i.e. 1, 2, 4 and 8 h) with either lithium (LiCl) or valproic acid (VA), since both drugs have been previously shown to modulate Wnt signaling via inhibition of GSK3β activity and thus increasing nuclear levels of β-catenin [34], [35]. Interestingly, we observed a marked enhancement of COX2 expression induced by 10–20 mM LiCl or 5–10 mM VA, that started soon after incubation with the compounds and which peaked after two hours of treatment (Figure 2A). Real time determination of COX2 mRNA levels in MKN45 cells similarly treated with LiCl or VA (2 h) indicated that COX2 was significantly up-regulated (Figure 2B) and that this effect was paralleled with the one observed on Wnt/β-catenin target genes c-myc [47] and cyclin D1 [48]. Next, in order to rule out the possibility that the observed phenomenon reflected non-specific effects of the drugs acting on proteins affecting different signaling cascades, we applied directly a fully functional purified Wnt3a protein to MKN45 cells (see Methods). These experiments clearly showed that 200–400 ng/ml of purified Wnt3a significantly enhanced COX2 mRNA expression after short periods of incubation (Figure 2), partially explaining the rapid effect of LiCl and VA and supporting a direct correlation between canonical Wnt/β-catenin activation and stimulation of COX-2 transcription in GC cells.

### Identification of novel TBE sites in the promoter of the COX2 gene

**Figure 3:**
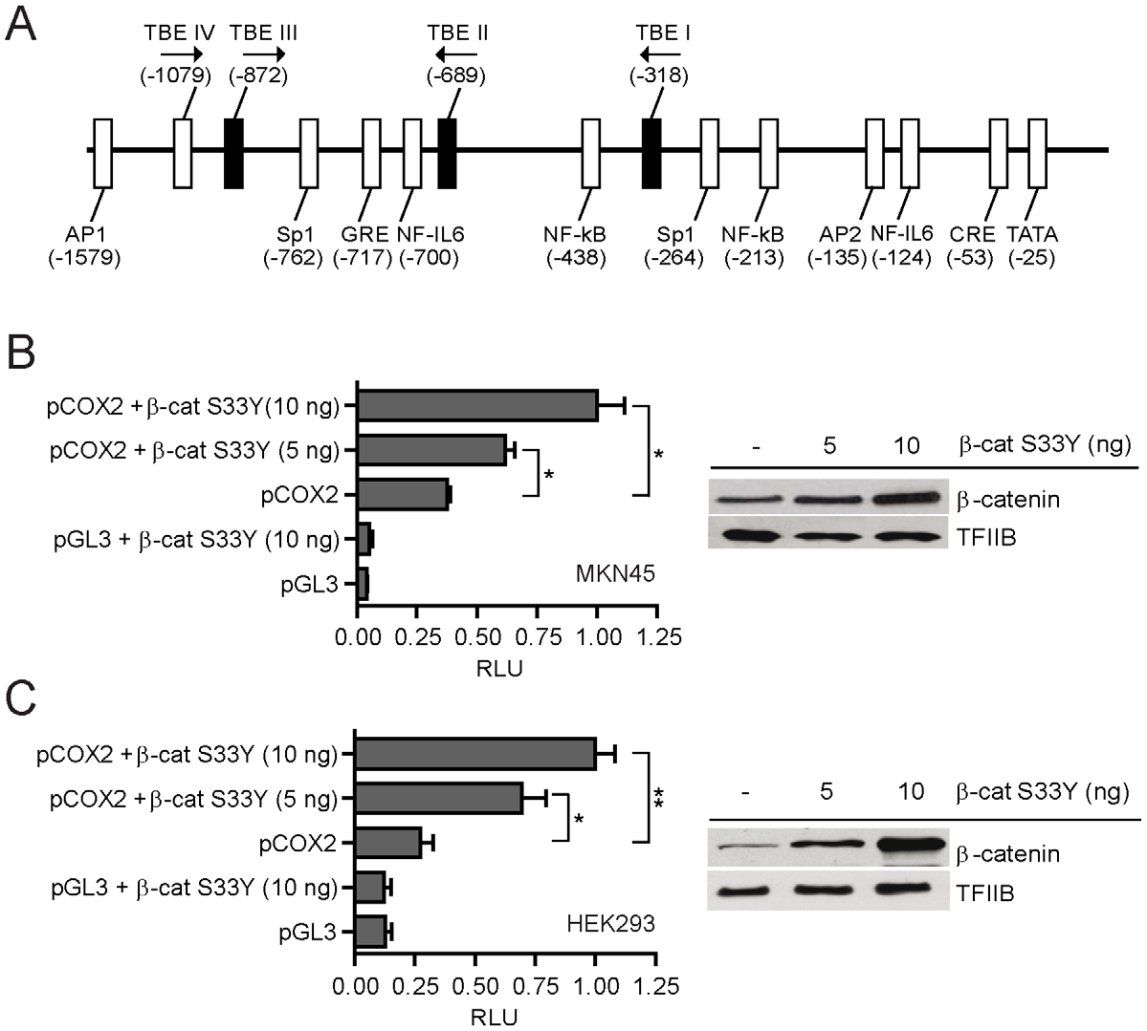
Human pCOX2-1.6 promoter activity in response to Wnt/β-catenin signaling. (A) Schematic drawing depicting the genomic context of ca. 1.6 Kbp from the transcriptional start site (TSS) of the human COX2 promoter, including the location of known transcriptional regulators (white boxes) and the position of the three novel TCF/LEF-binding elements (TBE: core CTTTG; black boxes) determined *in silico*. (B & C) Gene reporter assays in MKN45 (B) and HEK293 (C) cells co-transfected with 10 ng of pCOX2 and increasing concentrations of a constitutively active β-catenin (S33Y) protein (left panel). Cells were co-transfected with 1 ng of PRL-SV40 Renilla as an internal control. Promoter activity was normalized as the ratio between firefly luciferase and Renilla luciferase units. RLU: Relative Luciferase Units. Each figure corresponds to at least three independent experiments. Statistical significance was determined through ANOVA test (* p<0.05, ** p<0.01). Nuclear levels of β-catenin protein were examined in same cell lines through Western Blot analysis (right panel). The TFIIB general transcription factor was used as an internal control.

Previous studies indicated that a Wnt/β-catenin responsive TBE site (consensus core: CTTTG) is located at −1,079/−1,074 bp upstream from the transcriptional start site (TSS) of the human COX2 gene [21] (Site IV; Figure 3A, see also Figure S1). Further scanning of the 1,600 bp upstream sequence from the TSS [49] allowed us to identify 3 novel putative TBE sites: −877/−872 bp (Site III; sense orientation), −689/−684 bp and −318/−313 bp (Sites II and I, respectively; both in antisense orientation) (Figure 3A), which are not conserved between the human and murine COX2 genes (Figure S1). We therefore cloned the 1,600 bp segment from the human COX2 promoter (pCOX2), including all four TBE sites, into the pGL3-basic vector fused to the luciferase gene to be used subsequently in reporter assays (Figure 3A). Remarkably, paralleled transfection of 10 ng of this construct in MKN-45 and HEK293 cells revealed that pCOX2 displayed a significant higher (9-fold) basal promoter activity in MKN45 cells (Figure 3B and C). We hypothesized that this higher pCOX2 basal promoter activity could be related to the elevated content of nuclear β-catenin in this GC cell line (Figure 1B). Hence, MKN45 and HEK293 cells were co-transfected with pCOX2 in the presence of lower doses of a constitutive active β-catenin protein (S33Y) [31]. As observed in Figure 3, pCOX2 activity was indeed enhanced in both cell types, suggesting that β-catenin may bind and then activate TCF/LEF transcription factors at the functional TBE sites within the pCOX2 promoter.

### Contribution of TBE sites to COX2 promoter activity in GC cells

**Figure 4:**
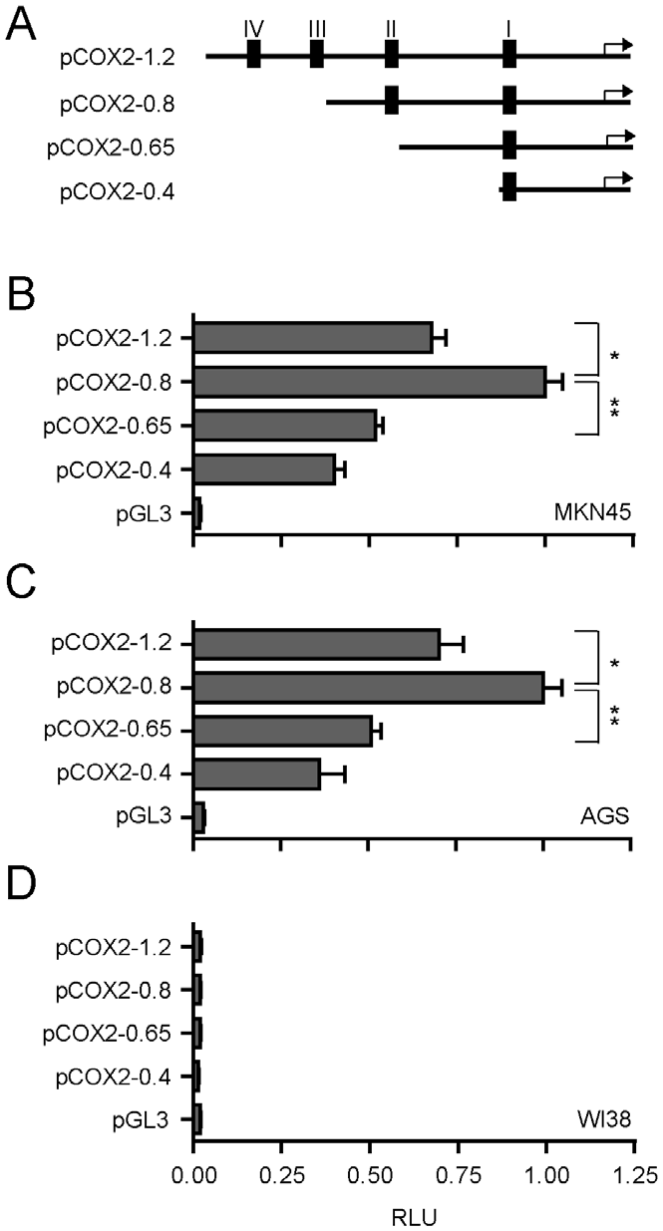
pCOX2-0.8 as a minimal COX2 promoter with maximum basal response in GC cells. (A) Schematic representation of pCOX2 deletions. (B–D) Gene reporter assay in MKN45 (B), AGS (C) and WI38 (D) cell lines transiently transfected with 50 ng pCOX deletions (pCOX2-1.2; pCOX2-0.8; pCOX2-0.65 and pCOX2-0.4) and 50 ng of empty vector. In all experiments cells were transfected with 1 ng of PRL-SV40 Renilla as an internal control. Promoter activity was normalized as the ratio between firefly luciferase and Renilla luciferase units. RLU: Relative Luciferase Units. Each figure corresponds to at least three independent experiments. Statistical significance was determined through ANOVA test (* p<0.05, ** p<0.01).

To dissect the contribution of Wnt/β-catenin responsive TBE sites on the transcriptional activity of the COX2 promoter four pCOX2 deletions constructs were generated: pCOX-1.2 (−1,123/+35 bp); pCOX-0.8 (−741/+35 bp); pCOX-0.65 (−582/+35 bp); and pCOX-0.4 (−371/+35 bp) (Figure 4A). These constructs were subsequently assayed for their activity through transient transfections in MKN45 and AGS cells, using the WI38 fibroblasts as a control cell line. Consistent with the endogenous COX2 expression levels in these GC cell lines (Figure 1A), pCOX2 deletions retained different levels of activity in MKN45 and AGS cells (Figure 4B and C). In contrast, no promoter activity was detected in WI38 fibroblasts (Figure 4D), even when the transfection doses in these cells were increased 8 fold (i.e. from 50 to 400 ng) (Figure S2). Importantly, and in agreement with previous reports [50], WI38 cells efficiently expressed a luciferase-reporter construct containing the promoter of the p21 gene (Figure S2), indicating that in these control cells the molecular machinery responsible for inducing COX2 transcription is not active. Further experiments revealed that transfection in MKN45 and AGS cells with the pCOX2-0.8 construct, which has 859 bp deleted from the 1.6 Kb pCOX2 reporter (Figure 4A), resulted in a significant increase in the promoter activity when compared with the pCOX2-1.2 reporter (Figure 4A–C). As no significant differences were observed between the 1.6 Kb pCOX2 and pCOX2-1.2 constructs (Figure S3), we did not perform further analyses with the larger construct. Importantly, pCOX2-0.8 represented the minimum size promoter fragment exhibiting the maximum basal activity, since further deletion of either 159 or 370 bp from the 5′-end, as it is the case with the pCOX2-0.65 or pCOX2-0.4 constructs, respectively, decreased more than 2-fold the levels of pCOX2 basal activity. These results indicate that the region comprised between 0.8 and 0.65 Kbp upstream of the TSS of the COX2 gene, and which includes a novel TBE response element (TBE Site II; −689/−684), may be a key component during the regulation of the basal level of COX2 gene expression in GC cells.

### Upregulation of pCOX2-0.8 via Wnt/β-catenin signaling

**Figure 5:**
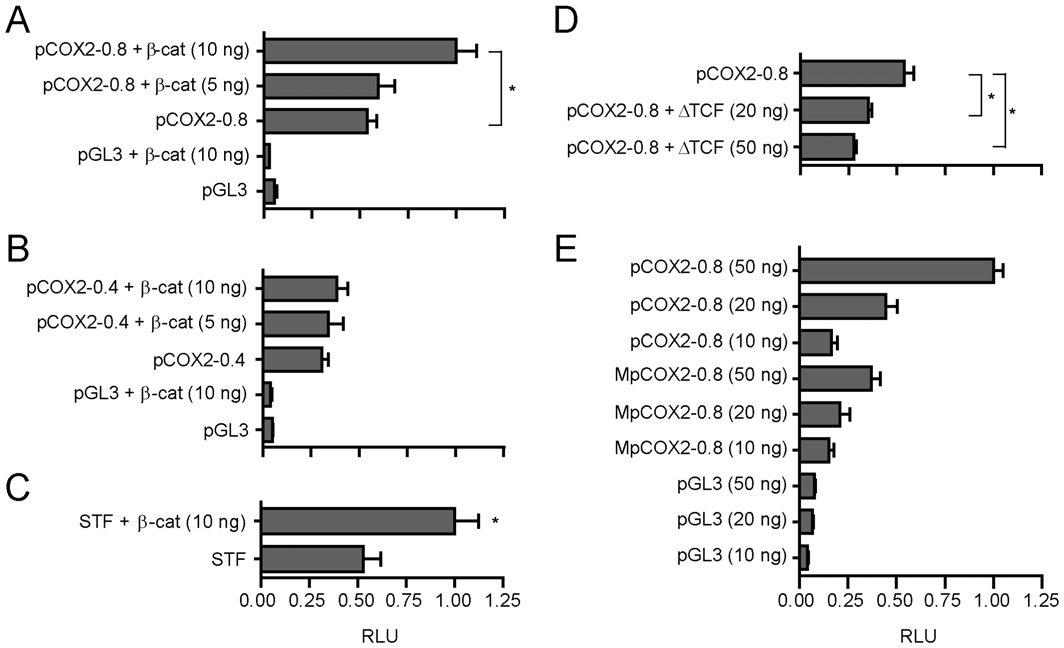
Wnt/β-catenin signaling modulates pCOX2-0.8 activity in MKN45 cells. Gene reporter assays in MKN45 cells transiently transfected with either 10 ng pCOX2-0.8 (A) or pCOX2-0.4 (B), plus 5–10 ng of β-catenin S33Y and 10 ng of empty vector as control. (C) SuperTOPFlash (STF; 10 ng) was co-transfected with 10 ng of β-catenin S33Y as a positive control for Wnt/β-catenin signaling activity. (D) Effect of a dominant negative TCF-4 (ΔTCF) construct on the activity of pCOX2-0.8. MKN45 cells were transiently co-transfected with 50 ng pCOX2-0.8 and 20–50 ng of ΔTCF. (E) Comparison between the activity of the wild-type pCOX2-0.8 and a mutant MpCOX2-0.8 construct, containing mutated residues in the TBE Site II in the pCOX2-0.8 promoter as background. Cells were transfected with increasing concentrations of pCOX2-0.8, MpCOX-08, or equal amounts of empty vector as a control. In all experiments 1 ng of PRL-SV40 Renilla was transfected as an internal control. Promoter activity was normalized as the ratio between firefly luciferase and Renilla luciferase units. RLU: Relative Luciferase Units. Each figure corresponds to at least three independent experiments. Statistical significance was determined through ANOVA test (* p<0.05, ** p<0.01).

We next examined the effect of Wnt/β-catenin signaling at the novel TBE Site II. We transfected MKN45 cells with the pCOX2-0.8 reporter construct and co-expressed the constitutive active β-catenin S33Y protein observing that there was a significant (2-fold) enhancement of the transcriptional activity of pCOX2-0.8. This β-catenin-mediated increase was specific since the pCOX2-0.4 construct was not stimulated in this β-catenin over-expression condition (Figure 5A and B). To control for Wnt/β-catenin signaling in MKN45 cells we used the Wnt/β-catenin responsive SuperTOPFLASH (STF) reporter carrying 12 copies of a TBE in tandem [32], which was transfected alone or in the presence of the plasmid coding for the mutant β-catenin S33Y protein. Interestingly, while co-expression with the mutant β-catenin S33Y protein in MKN45 cells was able to enhance (1.9-fold) the activity of the STF reporter, high levels of basal STF activity were detected in the absence of the mutant β-catenin (Figure 5C). This result is in agreement with our previous findings of high levels of endogenous nuclear β-catenin in this cell-type (see Figure 1B), and confirms that this nuclear factor is functional. To further confirm this finding, we performed identical experiments in HEK293 cells and obtained essentially similar results although in this case a clear dose-response curve was obtained when pCOX2-0.8 was transfected in the presence of increasing concentrations of the constitutively active β-catenin S33Y protein (Figure S4). Additionally, we found that STF activity in HEK293 cells was highly responsive to the levels of the exogenous mutant β-catenin (Figure S4C).

To further explore the effects of the Wnt/β-catenin pathway on the activity of the pCOX2-0.8 we carried out loss-of-function experiments with a dominant negative TCF4 (ΔTCF) construct, which codes for a transcription factor lacking 30 residues from its amino-terminus and that is unable to bind β-catenin [32]. We found that in MKN45 cells ΔTCF expression reduced the high levels of pCOX2-0.8 basal transcription in a dose-dependent manner (Figure 5D). This result confirms the key role of TCF/LEF transcription factors among the regulators of the activity of the pCOX2-0.8 promoter sequence, and further supports the idea that the TBE Site II (−689/−684) is involved in the response to Wnt/β-catenin signaling.

Finally, to directly address the role of the TBE Site II in Wnt/β-catenin-mediated regulation of the COX2 promoter, we introduced by site-directed mutagenesis a change in 2 key nucleotides of the consensus sequence of the TBE Site II core (i.e. pCOX2-0.8: CTTTG; MpCOX2-0.8: CCTCG). These changes have been shown previously to abolish Wnt/β-catenin-mediated transcription [39]. Transient transfection studies revealed that mutation of the TBE Site II significantly decreased (2–3 fold) the basal activity of pCOX2-0.8 construct in MKN45 cells (Figure 5E) when compared with the wild-type pCOX2-0.8 promoter construct. Moreover, and in agreement to our previous results, mutation of this TBE site II almost completely blocked β-catenin-mediated enhancement of the pCOX2-0.8 construct (Figure S4). Taken together, these results indicate that the TBE Site II (−689/−684) is a functional component in the COX2 gene transcriptional regulation.

### β-catenin binds to the COX2 gene promoter region containing the TBE Site II

Because a transcriptionally active conformation of chromatin structure is reflected by an elevated level of histone acetylation [51], we precipitated cross-linked chromatin fragments (average size 300–500 bp) isolated from MKN45 cells using polyclonal antibodies specific for acetylated histones H3 and H4. Similarly, we detected binding of RNA polymerase II (Pol II) as a parameter normally associated with transcriptional activity. We examined the enrichment in our precipitates of two promoter sequences: the proximal promoter (PP) region (−118/+62 from TSS) and the distal region (−793/−594) of the COX2 promoter containing the consensus TBE Site II (−689/−684). In particular, the TBE Site II was targeted since previous experiments indicated preferential binding of β-catenin to this promoter region (Figure S5). As a positive control, we evaluated binding at the TBE site in the promoter of the c-myc gene (−1,447/−1,144 from TSS), which was previously described in colon cancer cells [38].

**Figure 6:**
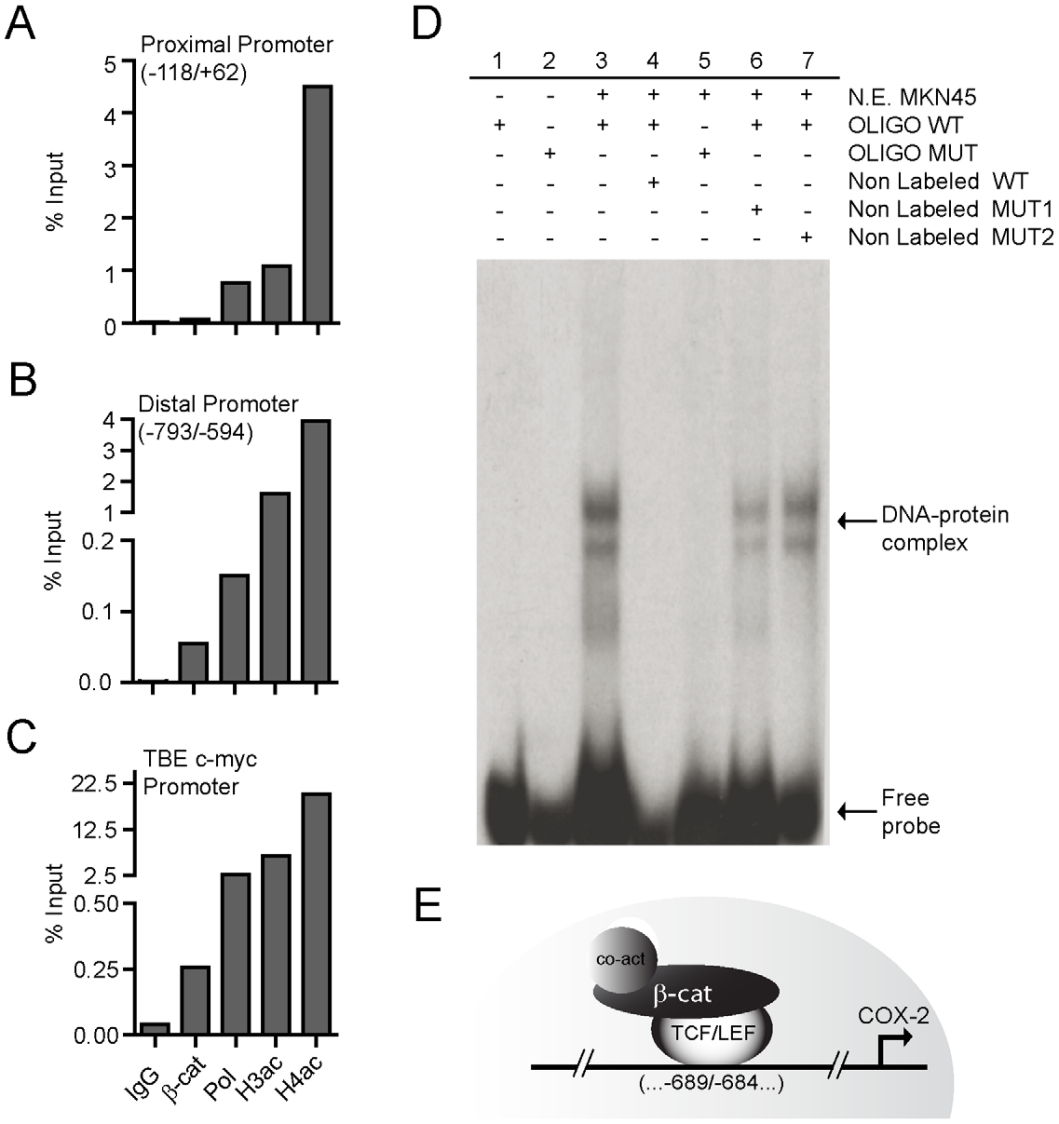
Binding of β-catenin to the TBE Site II (−689/−684) in the COX2 promoter. (A–C) ChIP assays in MKN45 cells using specific antibodies for β-catenin (β-cat), polymerase II (Pol), H3 and H4 acetylated histones (H3ac; H4ac) and immunoglobulin G (IgG). Quantification was done by real time PCR using specific primers for the proximal promoter (PP) region (A), the TBE Site II (−689/−684) in the human COX2 promoter (B) and a TBE site within the c-myc promoter, as a positive control (C). (D) EMSA assay in nuclear extracts from MKN45 cells. DNA-protein complexes formed by incubating nuclear extracts (N.E.) from MKN45 cells with radiolabelled probes containing the intact and a mutated TBE Site II (lanes 3 and 5) were resolved in native polyacrylamide gels at 5% and revealed through autoradiography. To determine the specificity of the binding a 50 times excess of non-radiolabelled wild-type (line 4) and mutant (6 and 7) oligonucleotides were added. Lanes 1 and 2 correspond to the wild-type and mutant radiolabelled oligonucleotides without incubation with N.E. These tests are representative of three independent experiments. E. Model depicting the molecular mechanism by which Wnt/β-catenin signaling may contribute to the expression of the human COX2 gene.

Acetylated histones H3 and H4 and the Polimerase II enzyme were found to bind within the proximal promoter region (Figure 6A), indicating that the chromatin structure around the COX2 promoter in MKN45 cells is in an open conformation, thus in agreement with our previous results demonstrating that the COX2 gene is actively transcribing. Notably, the β-catenin protein was immunoprecipitated from MKN45 samples mostly in association with the promoter region spanning the −684/−689 TBE sequence in the COX2 gene (Figure 6B). This factor was almost undetectable at the proximal promoter region, indicating that endogenous nuclear β-catenin is primarily recruited to the TBE Site II in these GC cells. As expected, endogenous β-catenin was similarly bound to the c-myc promoter TBE site, at levels that are comparable to those observed in the COX2 gene promoter (Figure 6C).

Collectively, these experiments indicate that the TBE Site II is directly involved in β-catenin-mediated transcriptional activation of the COX2 promoter. To further confirm these results we performed electrophoretic mobility shift assays (EMSA) in nuclear extracts of MKN45 cells. For this purpose we prepared 34 based-paired oligonucleotides; one containing the wild-type −689/−684 TBE Site II sequence (i.e. CTACAAAGA; residues underlined representing the core), and two oligonucleotides containing missense mutations in either the core of the TBE Site II (i.e. CTACGAGGA: TBE-MUT1) or in the flanking sequence (i.e. CGCCAAAGA: TBE-MUT2), as reported previously [21], [39]. As shown in Figure 6D, the radiolabeled wild-type TBE probe was capable to form retarded DNA-protein complexes when incubated with nuclear extracts from MKN45 cells. These protein-DNA complexes were specific since the addition of an excess of cold wild-type oligonucleotides (50-fold) dramatically inhibited the formation of the DNA-protein complex (Figure 6D, lane 3 and 4, respectively). The mutant radiolabeled TBE-MUT1 probe was neither able to form DNA-protein complexes on its own, nor to compete with the wild-type oligonucleotide when added in excess as a cold probe (Figure 6D, lane 5 and 6, respectively). Similar results were obtained when the second cold TBE-MUT2 probe was used as a competitor (Figure 6D, lane 7). Altogether, these results demonstrate that the novel TBE Site II in the COX2 promoter is involved in the transcriptional activation of the COX2 gene by recruiting the machinery responsible for transducing Wnt/β-catenin signaling (Figure 6E).

## Discussion

Previous studies have suggested a role for Wnt/β-catenin signaling during the onset and/or development of various types of cancer via modulating the expression of the COX2 gene [25]. In this work we have presented strong evidence supporting a direct role for Wnt/β-catenin signaling in the control of COX2 expression in GC cells. First, we have shown that there is a tight correlation between COX2 expression and the nuclear content of β-catenin, which seems to be independent of either malignancy or transformation state of CG cells. Second, time- and dose-dependent enhancement of COX2 expression was observed soon after induction (2 h) with either pharmacological compounds mimicking Wnt/β-catenin signaling. Third, gene reporter assays evaluating the COX2 promoter activity in response to gain- and loss-of-function experiments, including a constitutively active β-catenin protein and dominant negative TCF4 transcription factor, demonstrate that Wnt/β-catenin components are involved in the transcriptional regulation of the COX2 gene.

We have also shown here that within 2 Kbp upstream of the human COX2 promoter there are four putative TBE sites (core: CTTTG), one of which has been previously studied by Araki and cols [21], [28]. Through gene reporter assays with different pCOX2 deletion constructs we showed that pCOX2-0.8 displayed the highest basal transcriptional activity in the GC cells MKN45 and AGS. Interestingly, pCOX2-0.8 maintained the TBE Site-II (−689/−684) integrity indicating its key contribution to the regulation of the transcriptional activity of the COX2 gene. Notably, the complete pCOX2 TBE Site II signature (i.e. 5′-WWCAAAGS-3′; S = C/G; W = A/T), resembles the optimal TCF site described by van de Wetering and cols. [52], which was subsequently used to identify genuine Wnt-transcriptional targets in the *Drosophila* genes *nkd* and *CG6234* [53].

Functionality of this TBE Site II was also confirmed by our mutagenesis studies. Thus, when we mutated the TBE-signature sequence into the pCOX2-0.8 construct (MpCOX2-0.8), it was found that basal COX2 promoter activity was significantly affected in MKN45 cells. In addition our ChIP and EMSA analyses confirmed that β-catenin is preferentially recruited to the −689/−684 COX2 promoter region in GC cells, at a level that resembles that found at the c-myc promoter [38]. Such interaction preferentially occurs on this region of the COX2 gene promoter as no β-catenin is found to bind to the proximal promoter region of the COX2 gene. Altogether these results indicate that the TBE Site II is a functional Wnt/β-catenin responsive element within the human COX2 promoter. Our data, nevertheless, does not rule out the contribution of other genomic regions in the human COX2 gene [21]. Instead, we propose that in GC cells a complex web of interactions is present where the TBE Site II cooperates with other response elements to up-regulate COX2 expression.

As our results in GC cells are in agreement with those reported for colon cancer cells, it is tempting to speculate that Wnt/β-catenin signaling could be similarly involved in COX2 regulation in other tumors [21], [28]. Indeed, moderate to strong protein levels of β-catenin can be observed in ca. 72% of all cancers analyzed in the Human Atlas Protein tissue database [54]. Similarly, strong to moderate cytoplasmic COX2 staining and occasional membranous reactivity is observed in 50% of all cancers, including colorectal, prostate, cervical, endometrial, urothelial, pancreatic, liver and in glandular cells from gastric tumor tissue. Interestingly, simultaneous detection of COX2 and β-catenin immunoreactivity in these GC tumors is observed in 8 out of 12 identical individuals (Figure S6 and Table S2), arguing in favor of a positive relationship between COX2 expression and the intracellular levels of the β-catenin protein, and thus the activity of the canonical Wnt signaling pathway in the transcriptional regulation of the COX2 gene.

## Supporting Information

Figure S1 **ClustalW multiple sequence alignment of human and murine COX2 promoters.** TBE response elements, as well as recognition sequences for SP1 and CRE transcription factors, and the TATA box are enclosed.
(PDF)

Figure S2 **Comparison of basal promoter activity of pCOX2-1.6 and pCOX2-1.2 constructs in MKN45 and HEK293 cells.** Gene reporter assays in MKN45 (A) and HEK293 (B) cells transiently transfected with increasing concentrations of pCOX2-1.6 or pCOX2-1.2. In all experiments 1 ng of PRL-SV40 Renilla was transfected as an internal control. Promoter activity was normalized as the ratio between firefly luciferase and Renilla luciferase units. RLU: Relative Luciferase Units. Each figure corresponds to at least three independent experiments. Statistical significance was determined through ANOVA test (* p<0.05, ** p<0.01).
(PDF)

Figure S3 **Basal promoter activity of pCOX2-deletion constructs examined in WI38 cells.** Wi38 cells were transiently transfected with 400 ng pCOX2-deletion constructs (A) or a construct containing the p21 promoter as a control (B) and basal promoter activity was determined. In all experiments 1 ng of PRL-SV40 Renilla was transfected as an internal control. Promoter activity was normalized as the ratio between firefly luciferase and Renilla luciferase units. RLU: Relative Luciferase Units. Each figure corresponds to at least three independent experiments. Statistical significance was determined through ANOVA test (* p<0.05, ** p<0.01).
(PDF)

Figure S4 **pCOX2-0.8 activity in response to Wnt/β-catenin signaling in HEK293 cells.** (A) Reporter gene assay HEK293 cells, co-transfected transiently with 10 ng pCOX-0, 8 with 5 and 10 ng of β-catenin S33Y and 10 ng of empty vector as control. (B) MKN45 cells was transiently transfected with 10 ng pCOX2-0,4 with 5 and 10 ng of S33Y β-catenin and 10 ng of empty vector as control. (C) As a positive control 10 ng of Super Top Flash (STF) was co-transfected with 10 ng of S33Y β-catenin. (D) Effect of site-directed mutation in TBE site in pCOX2-0,8 reporter gene assays in HEK293 cells transfected with 10 ng of pCOX2-0,8 and mutated pCOX2-0,8 (MpCOX-08) in the presence and absence of 5 and 10 ng of S33Y β-catenin, using equal amounts of empty vector as a control. In all trials 1 ng of PRL-SV40 Renilla was transfected as an internal control. Promoter activity was normalized as the ratio between firefly luciferase and Renilla units (RLU). Each figure corresponds to a representative result of three independent experiments. Statistical significance was determined through ANOVA test (* p<0.05, ** p<0.01).
(PDF)

Figure S5 **Binding of β-catenin to TBE sites in the COX-2 promoter.** ChIP assays in MKN45 cells using specific antibodies for β-catenin. Quantification was performed by real time PCR using specific primers to TBE IV site (−1079/−1074), TBE III site (−877/872), TBE II (−689/−684) and TBE I (−318/−313) and normalizated by IgG.
(PDF)

Figure S6 **COX2 and β-catenin immunoreactivity in gastric cancer tissue.** Immunohistochemical images of gastric cancer samples showing moderate to strong levels of COX2 and β-catenin expression in different individuals (ID; see Table S2 for details) analyzed as part of the Human Protein Atlas initiative (http://www.proteinatlas.org). COX2 and β-catenin proteins were visualized using the HPA001335 and the CAB000108 antibodies, respectively.
(PDF)

Table S1 **Primers used in this study.**
(PDF)

Table S2 **Gastric cancer samples analysed for COX2 and -catenin levels in the Human Atlas Protein Collection.**
(PDF)
