# Supplementary material for: Wnt/β-Catenin Signaling Enhances Cyclooxygenase-2 (COX2) Transcriptional Activity in Gastric Cancer Cells
Source: PLoS One. 2011 Apr 6;6(4):e18562. doi: 10.1371/journal.pone.0018562 (PMC3071840; doi:10.1371/journal.pone.0018562)
Supplement: Figure S1 — ClustalW multiple sequence alignment of human and murine COX2 promoters. TBE response elements, as well as recognition sequences for SP1 and CRE transcription factors, and the TATA box are enclosed. (PDF) [file pone.0018562.s001.pdf]

```
Mm   ACTGTCCTCAGAATATAGTGTAATTTCACACTCTGTGTCATTTTTTTTA-TCCATTCACG -1074
Rn   ACTTTCCTGAGAATACAGTTTAATTTCAGATTTTGTGTCTTTTTTTTTTCTCCATTCATG -1084
Hs   ACTTTGATC----CATGGTCACAACTCATAATCTTGGAAAAGTGGA------CAGAAAAG -1032
     *** *  *       *  **   *  *** * * *  *      *         **   * *
       TBE IV

Mm   ATATCCAACACTATCAAAAACATCACCTCTCTAGGCAAATAATTTTT--TATCAAACACT -903
Rn   TTATTCAACAGTCAAAAAAAAATCACCTCTCTAGGCAATTAATTTTTATTATCAAGCAAT -907
Hs   ACATTTAGCGTCCCTGCAAATTCTGGCCATC---GCCGCTTCCTT----TGTCCA----- -868
       **  * *        ***      *  **    **    *   **      * ** *
                                                  TBE III

Mm   CGTGAAAAGAGTTGCTGATCAAAATGA-TAAAAGCTATGTAACAGCAGGG-GGAAAATAC -676
Rn   TGTGAAA-GAGTTGTTGATCAAAATGA-TAAAAGCTATGTAACAGCAGGGAGGAAAATAC -674
Hs   ----AAGCAACTTAGCTACAAAGATAAATTACAGCTATGTA-CACTGAAG--GTAGCTAT -651
         **   * **    *  **  ** * * * ********* **      *  * *  **
                     TBE II

Mm   TTAGATCCCGGGAGGGGAAGCTGTGACA--CTCTTGAGCTTTTAGGCCCCCACTGGATGC -326
Rn   TCGGATCCCGGGAGGGGAAGCTGTGACATTCTCTTGCTCCTCCGGCCCCCCAGTGGATGC -326
Hs   CGGTATCCCATCCAAGGCGATCAGTCCAG-AACTGGCTCTCGGAAGCGCTCGGGCAA--- -316
         *****      **          **     ** *  *        * * *    *
                                                               TBE I

Mm   GCGACTGGGAGGAAACCGGAGACCCCAAAGAGCGCCAGACTAGGCGCAGACTCAGCGAAC -266
Rn   GGGACTGGGAGGAAACCCGAGACCTCAAAGAGAGCCAGTCTTGGAGCAGGCACAGCGAAC -266
Hs   -AGACTGCGAAGAAGAAAAGACATCTGGCGGAAACCTG---TGCGCCTGGGGCGGTGGAA -260
       ***** ** ***                *     ** *    *    * *   * * * *
                                                         Sp1

                          Sp1
Mm   CACAGGGCGCCTGGAGGGATGGAGAGGGCGGTGCAGCTCTC-TTGGCACCACCTGGGGCA -207
Rn   CACAGGGCGCCTGGAAGGATGCAGAGGGCGGTGCAGCTCTC-TTGGCACCACTTTGGGCA -207
Hs   CTCGGGGAGG--AGAGGGAGGGATCAGACAGGAGAGTGGGGACTACCCCCTCTGCTCCCA -202
     * * *** *    ** *** * *    * * *    **        *  * ** *      **

Mm   GCCAAGGGCAGCTTCCCGGCTTCCTTCGTCTCTCATTTGCGTGGGTAAAAG--CCTGCCG -149
Rn   GCCGAGGGAAGCTTCCTGGCTTCTCTGGGCTC--ATTTGCGTGAGTAAA-G--CCTGCCC -151
Hs   AATTGGGGCAGCTTCCTGGGTTTCCGATTTTCTCATTTCCGTGGGTAAAAAACCCTGCCC -142
          *** ******* ** **        **  **** **** *****    ******
                                            CRE
Mm   TTGGGGAAAGCCTAAGCGGAAAGACAGAGTCACCACTACGTCACGTGGAGTCCGCTTTAC -35
Rn   GTGGGGAAAGCCGAGGCGGAAAGACACAGTCACGAA---GTCACGTGGAGTCCACTTTAC -37
Hs   GTACGAAAAG-----GCGGAAAGAAACAGTCATTTC---GTCACATGGGCTTGGTTTTCA -36
      *  * ****     ********* * *****        ***** ***  *    ***
                                       CRE
Mm   --AGACTTAAAAGCAAGGTTCTCCCCATTAGCAGCC                         +1
Rn   TAAGATTTAAAAGCAAGGTTCTCCGGGTTAGCGGCC                         +1
Hs   GTCTTATAAAAAGGAAGGTTCTCTCGGTTAGCGACC                         +1
         * ***** *********     *****  **
       TATA box
```

Supplemental Fig. S1. ClustalW multiple sequence alignment of human and murine COX2 promoters. TBE response elements, as well as recognition sequences for SP1 and CRE transcription factors, and the TATA box are enclosed.
