# Supplementary material for: Wnt/β-Catenin Signaling Enhances Cyclooxygenase-2 (COX2) Transcriptional Activity in Gastric Cancer Cells
Source: PLoS One. 2011 Apr 6;6(4):e18562. doi: 10.1371/journal.pone.0018562 (PMC3071840; doi:10.1371/journal.pone.0018562)
Supplement: Figure S2 — Comparison of basal promoter activity of pCOX2-1.6 and pCOX2-1.2 constructs in MKN45 and HEK293 cells. Gene reporter assays in MKN45 (A) and HEK293 (B) cells transiently transfected with increasing concentrations of pCOX2-1.6 or pCOX2-1.2. In all experiments 1 ng of PRL-SV40 Renilla was transfected as an internal control. Promoter activity was normalized as the ratio between firefly luciferase and Renilla luciferase units. RLU: Relative Luciferase Units. Each figure corresponds to at least three independent experiments. Statistical significance was determined through ANOVA test (* p<0.05, ** p<0.01). (PDF) [file pone.0018562.s002.pdf]

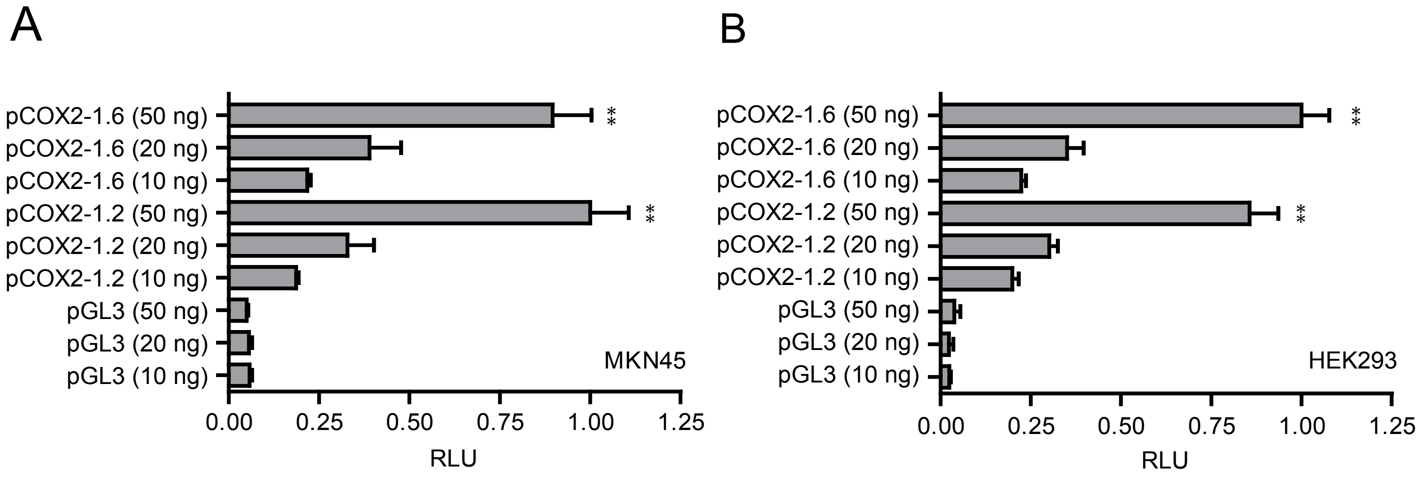

<u>Supplemental Fig. S2</u>. Comparison of basal promoter activity of pCOX2-1.6 and pCOX2-1.2 constructs in MKN45 and HEK293 cells. Gene reporter assays in MKN45 (*A*) and HEK293 (*B*) cells transiently transfected with increasing concentrations of pCOX2-1.6 or pCOX2-1.2. In all experiments 1 ng of PRL-SV40 Renilla was transfected as an internal control. Promoter activity was normalized as the ratio between firefly luciferase and Renilla luciferase units. RLU: Relative Luciferase Units. Each figure corresponds to at least three independent experiments. Statistical significance was determined through ANOVA test (* $p < 0.05$, ** $p < 0.01$).
