# Supplementary material for: Wnt/β-Catenin Signaling Enhances Cyclooxygenase-2 (COX2) Transcriptional Activity in Gastric Cancer Cells
Source: PLoS One. 2011 Apr 6;6(4):e18562. doi: 10.1371/journal.pone.0018562 (PMC3071840; doi:10.1371/journal.pone.0018562)
Supplement: Figure S3 — Basal promoter activity of pCOX2-deletion constructs examined in WI38 cells. Wi38 cells were transiently transfected with 400 ng pCOX2-deletion constructs (A) or a construct containing the p21 promoter as a control (B) and basal promoter activity was determined. In all experiments 1 ng of PRL-SV40 Renilla was transfected as an internal control. Promoter activity was normalized as the ratio between firefly luciferase and Renilla luciferase units. RLU: Relative Luciferase Units. Each figure corresponds to at least three independent experiments. Statistical significance was determined through ANOVA test (* p<0.05, ** p<0.01). (PDF) [file pone.0018562.s003.pdf]

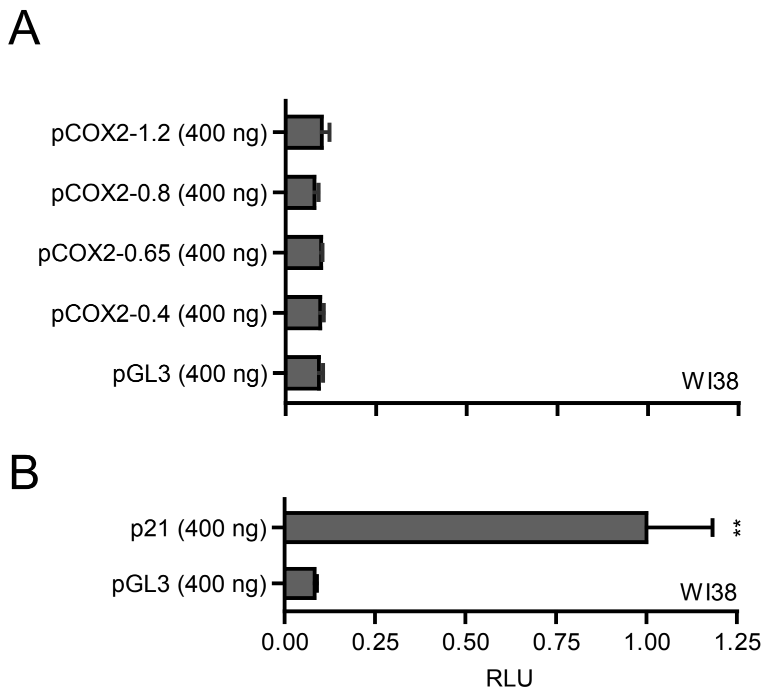


<u>Supplemental Fig. S3</u>. Basal promoter activity of pCOX2-deletion constructs examined in WI38 cells. Wi38 cells were transiently transfected with 400 ng pCOX2-deletion constructs (*A*) or a construct containing the p21 promoter as a control (*B*) and basal promoter activity was determined. In all experiments 1 ng of PRL-SV40 Renilla was transfected as an internal control. Promoter activity was normalized as the ratio between firefly luciferase and Renilla luciferase units. RLU: Relative Luciferase Units. Each figure corresponds to at least three independent experiments. Statistical significance was determined through ANOVA test (* $p < 0.05$, ** $p < 0.01$).
