# Supplementary material for: Wnt/β-Catenin Signaling Enhances Cyclooxygenase-2 (COX2) Transcriptional Activity in Gastric Cancer Cells
Source: PLoS One. 2011 Apr 6;6(4):e18562. doi: 10.1371/journal.pone.0018562 (PMC3071840; doi:10.1371/journal.pone.0018562)
Supplement: Figure S4 — pCOX2-0.8 activity in response to Wnt/β-catenin signaling in HEK293 cells. (A) Reporter gene assay HEK293 cells, co-transfected transiently with 10 ng pCOX-0, 8 with 5 and 10 ng of β-catenin S33Y and 10 ng of empty vector as control. (B) MKN45 cells was transiently transfected with 10 ng pCOX2-0,4 with 5 and 10 ng of S33Y β-catenin and 10 ng of empty vector as control. (C) As a positive control 10 ng of Super Top Flash (STF) was co-transfected with 10 ng of S33Y β-catenin. (D) Effect of site-directed mutation in TBE site in pCOX2-0,8 reporter gene assays in HEK293 cells transfected with 10 ng of pCOX2-0,8 and mutated pCOX2-0,8 (MpCOX-08) in the presence and absence of 5 and 10 ng of S33Y β-catenin, using equal amounts of empty vector as a control. In all trials 1 ng of PRL-SV40 Renilla was transfected as an internal control. Promoter activity was normalized as the ratio between firefly luciferase and Renilla units (RLU). Each figure corresponds to a representative result of three independent experiments. Statistical significance was determined through ANOVA test (* p<0.05, ** p<0.01). (PDF) [file pone.0018562.s004.pdf]

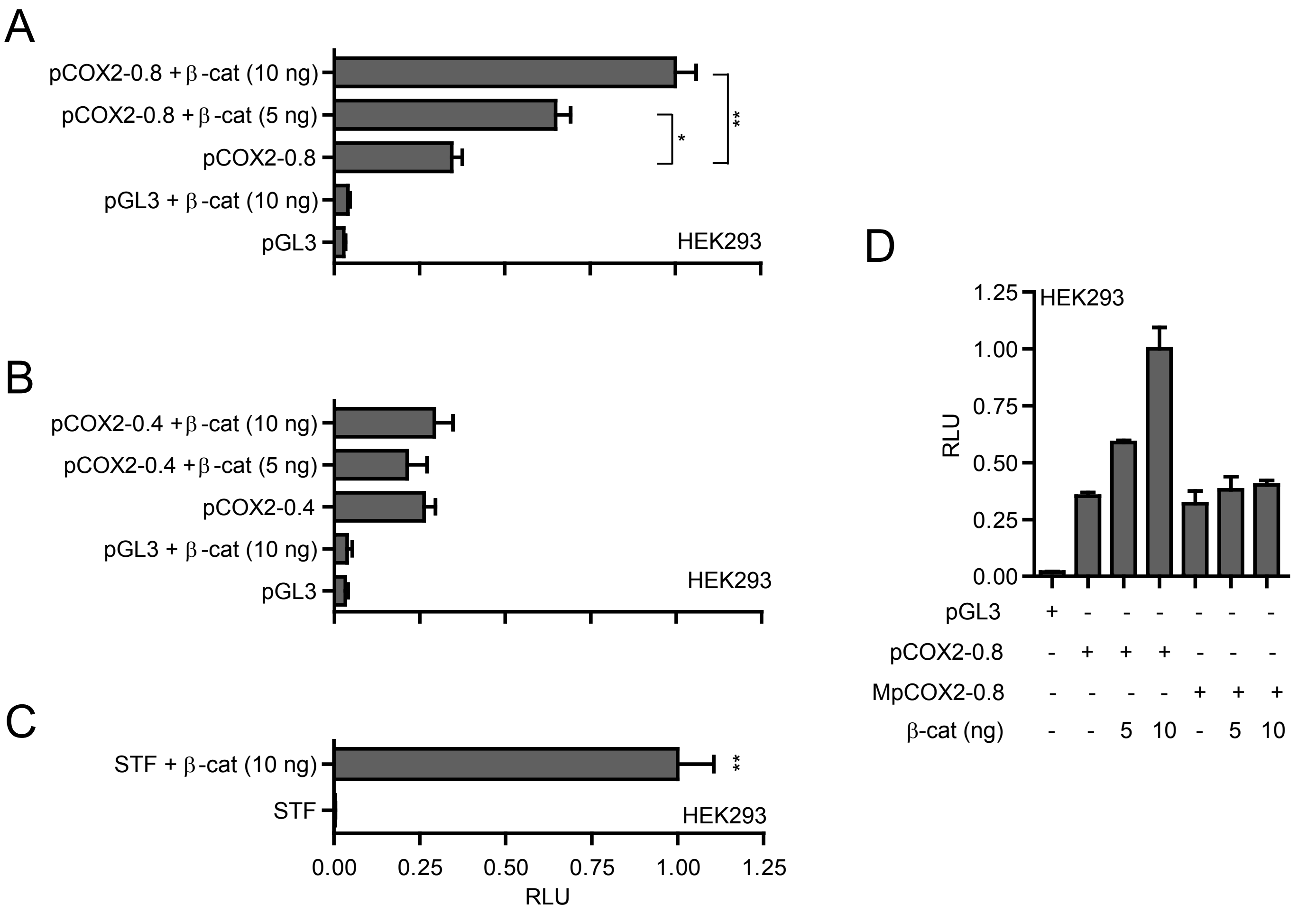

Supplemental Fig. S4. pCOX2-0.8 activity in response to Wnt/β-catenin signaling in HEK293 cells. *(A)* Reporter gene assay HEK293 cells, co-transfected transiently with 10 ng pCOX-0, 8 with 5 and 10 ng of β-catenin S33Y and 10 ng of empty vector as control. (*B)* MKN45 cells was transiently transfected with 10 ng pCOX2-0,4 with 5 and 10 ng of S33Y β-catenin and 10 ng of empty vector as control. (*C*) As a positive control 10ng of Super Top Flash (STF) was co-transfected with 10 ng of S33Y β-catenin. (*D)* Effect of site-directed mutation in TBE site in pCOX2-0,8 reporter gene assays in HEK293 cells transfected with 10 ng of pCOX2-0,8 and mutated pCOX2-0,8 (MpCOX-08) in the presence and absence of 5 and 10 ng of S33Y β-catenin, using equal amounts of empty vector as a control. In all trials 1 ng of PRL-SV40 Renilla was transfected as an internal control. Promoter activity was normalized as the ratio between firefly luciferase and Renilla units (RLU). Each figure corresponds to a representative result of three independent experiments. Statistical significance was determined through ANOVA test (* p <0.05, ** p <0.01).
