# Supplementary material for: Wnt/β-Catenin Signaling Enhances Cyclooxygenase-2 (COX2) Transcriptional Activity in Gastric Cancer Cells
Source: PLoS One. 2011 Apr 6;6(4):e18562. doi: 10.1371/journal.pone.0018562 (PMC3071840; doi:10.1371/journal.pone.0018562)
Supplement: Figure S5 — Binding of β-catenin to TBE sites in the COX-2 promoter. ChIP assays in MKN45 cells using specific antibodies for β-catenin. Quantification was performed by real time PCR using specific primers to TBE IV site (−1079/−1074), TBE III site (−877/872), TBE II (−689/−684) and TBE I (−318/−313) and normalizated by IgG. (PDF) [file pone.0018562.s005.pdf]

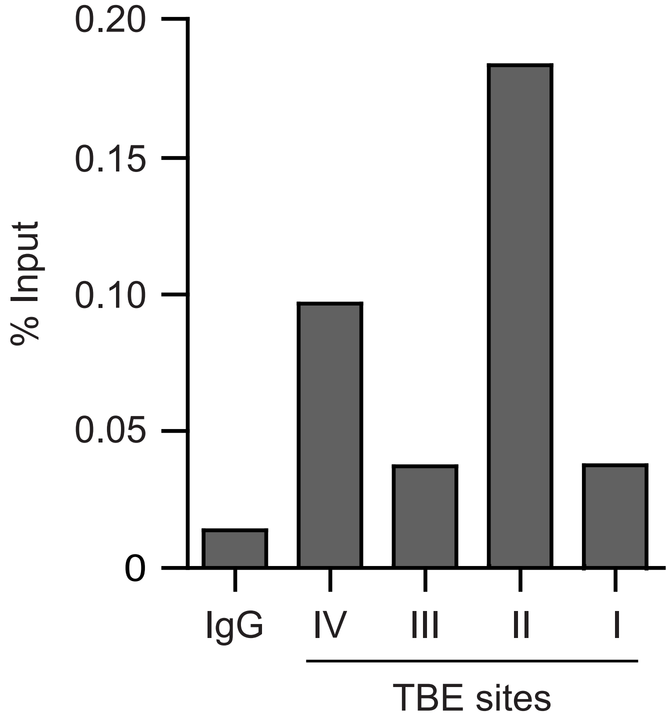

<u>Supplemental Fig. S5</u>. Binding of β-catenin to TBE sites in the COX-2 promoter. ChIP assays in MKN45 cells using specific antibodies for β-catenin. Quantification was performed by real time PCR using specific primers to TBE IV site (-1079/-1074), TBE III site (-877/872), TBE II (-689/-684) and TBE I (-318/-313) and normalizated by IgG.
