# Supplementary material for: Wnt/β-Catenin Signaling Enhances Cyclooxygenase-2 (COX2) Transcriptional Activity in Gastric Cancer Cells
Source: PLoS One. 2011 Apr 6;6(4):e18562. doi: 10.1371/journal.pone.0018562 (PMC3071840; doi:10.1371/journal.pone.0018562)
Supplement: Figure S6 — COX2 and β-catenin immunoreactivity in gastric cancer tissue. Immunohistochemical images of gastric cancer samples showing moderate to strong levels of COX2 and β-catenin expression in different individuals (ID; see Table S2 for details) analyzed as part of the Human Protein Atlas initiative (http://www.proteinatlas.org). COX2 and β-catenin proteins were visualized using the HPA001335 and the CAB000108 antibodies, respectively. (PDF) [file pone.0018562.s006.pdf]

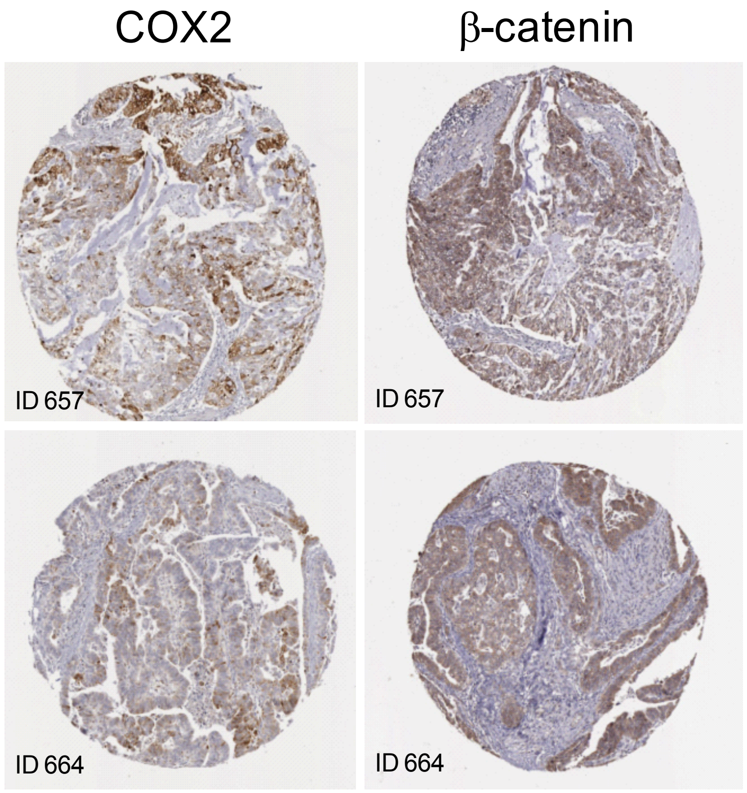

Supplemental Fig. S6. COX2 and β-catenin immunoreactivity in gastric cancer tissue. Immunohistochemical images of gastric cancer samples showing moderate to strong levels of COX2 and β-catenin expression in different individuals (ID; see Supplemental Table 2 for details) analyzed as part of the Human Protein Atlas initiative (http://www.proteinatlas.org). COX2 and β-catenin proteins were visualized using the HPA001335 and the CAB000108 antibodies, respectively.
