# Supplementary material for: Wnt/β-Catenin Signaling Enhances Cyclooxygenase-2 (COX2) Transcriptional Activity in Gastric Cancer Cells
Source: PLoS One. 2011 Apr 6;6(4):e18562. doi: 10.1371/journal.pone.0018562 (PMC3071840; doi:10.1371/journal.pone.0018562)
Supplement: Table S1 — Primers used in this study. (PDF) [file pone.0018562.s007.pdf]

Supplemental Table S1. Primers used in this study.

COX2-promoter constructs (Luciferase assays)

Promoter construct	Forward primer 5’-3’	Reverse primer 5’-3’
pCOX2	AAACTCGACAATAAATAGGAGTGCCATAAATG	TGCTCCTGACGCTCACTGCAAAAGCTTA
pCOX2-1.2	AAACTCGAGGCCCCAATAAGCCCAGGCAA	TGCTCCTGACGCTCACTGCAAAAGCTTA
pCOX2-0.8	ACACTCGAGGGGTGAAGGTCAGGAGAACA	TGCTCCTGACGCTCACTGCAAAAGCTTA
pCOX2-0.65	TGAAGGTAGCTATTTCATTCCACA	TGCTCCTGACGCTCACTGCAAAAGCTTA
pCOX2-0.4	GCGAAGAAGAAAAGACATCTGG	TGCTCCTGACGCTCACTGCAAAAGCTTA

Site directed mutagenesis

Name	Forward primer 5’-3’	Reverse primer 5’-3’
pCOX2-0.8-TBEMUT	XGAAAGCAACTTA**GTA*CC* AAG**ATAAATTACAGC	GCTAATTTAATCC**CTT*GG* AC**TAAGTTGCTTTC

ChIP Q-PCR

Name	Forward primer 5’-3’	Reverse primer 5’-3’
ChIP-TBECOX2-I	GAAGCCAAGTGTCCTTCTGC	GGAGAGGGAGGGATCAGAC
ChIP-TBECOX2-II	CGAGAATAGAAAATTAGCCCCAATAAGC	TTTTGTGGAATGAAATAGCTACC
ChIP-TBECOX2-III	AAGGCATACGTTTTGGACATTTAGC	CTTTATATTGGTGACCCGTGGAGCT
ChIP-TBECOX2-IV	CGAGAATAGAAAATTAGCCCCAATAAGC	CAACTCATAATCTTGGAAAAGTGGACA
ChIP-TBECMYC	GTGAATACACGTTTGCGGGTTAC	AGAGACCCTTGTGAAAAAAACCG
ChIP-COX2POLII	AGGCGCTGCTGAGGAGTT	TAAGGGGAGAGGAGGGAAAA

RT-PCR

Name	Forward primer 5’-3’	Reverse primer 5’-3’
RT-COX2	AAGCCTTCTCTAACCTCT	GTGAATACACGTTTGCGGGTTAC

Real Time-PCR

Name	Forward primer 5’-3’	Reverse primer 5’-3’
Real-COX2	TCAAATGAGATTGTGGAAAAAT	AGATCATCTCTGCCTGAGTATCTT
Real-C-MYC	TACCCTCTCAACGACAGCAG	TCTTGACATTCTCCTCGGTG
Real-CCND1	GCCGAGAAGCTGTGCATCTA	CTGGCATTTTGGAGAGGAAG
Real-β–actin	AGAAAATCTGGCACCACACC	CAGAGGCGTACAGGGATAGC

EMSA

Oligo name	Forward Oligo sequence 5’-3’
COX2-TBEwt	AAAGCAACTTAG**CTACAAAGA**TAAATTACAGC
COX2-TBEmut	AAAGCAACTTAG**CTACGAGGA**TAAATTACAGC
COX2-TBEmut II	AAAGCAACTTAG**CGCCAAAGA**TAAATTACAGC
