# Supplementary material for: Wnt/β-Catenin Signaling Enhances Cyclooxygenase-2 (COX2) Transcriptional Activity in Gastric Cancer Cells
Source: PLoS One. 2011 Apr 6;6(4):e18562. doi: 10.1371/journal.pone.0018562 (PMC3071840; doi:10.1371/journal.pone.0018562)
Supplement: Table S2 — Gastric cancer samples analysed for COX2 and -catenin levels in the Human Atlas Protein Collection. (PDF) [file pone.0018562.s008.pdf]

Supplemental Table S2. Gastric cancer samples analysed for COX2 and β-catenin levels in the Human Atlas Protein Collection

Patient ID	Sex	AGE	COX2[1]	Staining	β-catenin[2]	Staining
1186	m	78	< 25%	cytoplasmic/membrane	> 75%	cytoplasmic/membrane
893	m	77	---	---	> 75%	cytoplasmic/membrane
657	f	52	< 25%	cytoplasmic/membrane	> 75%	cytoplasmic/membrane
703	m	71	---	---	> 75%	cytoplasmic/membrane
198	m	80	< 25%	cytoplasmic/membrane	25-75%	cytoplasmic/membrane
1073	m	76	> 75%	cytoplasmic/membrane	< 25%	cytoplasmic/membrane
1266	m	47	< 25%	cytoplasmic/membrane	25-75%	cytoplasmic/membrane
664	f	50	< 25%	cytoplasmic/membrane	25-75%	cytoplasmic/membrane
1158	m	88	---	---	25-75%	cytoplasmic/membrane
549	f	86	---	---	25-75%	cytoplasmic/membrane
1207	m	63	< 25%	cytoplasmic/membrane	25-75%	cytoplasmic/membrane
2645	m	74	< 25%	cytoplasmic/membrane	25-75%	cytoplasmic/membrane

[1]PTGS2: HPA001335 Abody

[2]CTNNB1: CAB000108 Abody
